# Seroprevalence of hepatitis E virus (HEV) among male craft and manual workers in Qatar (2020–2021)

**DOI:** 10.1016/j.heliyon.2023.e21404

**Published:** 2023-10-31

**Authors:** Nadin Younes, Hadi M. Yassine, Parveen Banu Nizamuddin, Katerina Kourentzi, Patrick Tang, Houssein H. Ayoub, Makiyeh Khalili, Peter V. Coyle, Dmitri Litvinov, Richard C. Willson, Laith J. Abu-Raddad, Gheyath K. Nasrallah

**Affiliations:** a Biomedical Research Center, Qatar University, Doha, 2713, Qatar; b Department of Biomedical Science, College of Health Sciences, Member of QU Health, Qatar University, Doha, 2713, Qatar; c William A. Brookshire Department of Chemical and Biomolecular Engineering, University of Houston, Houston, TX 77204, USA; d Division of Microbiology, Sidra Medicine, Doha, 26999, Qatar; e Mathematics Program, Department of Mathematics, Statistics, and Physics, College of Arts and Sciences, Qatar University, Doha, 2713, Qatar; f Department of Laboratory Medicine, Hamad Medical Corporation, Doha, 3050, Qatar; g Department of Pediatrics, Women's Wellness and Research Center, Hamad Medical Corporation, Doha, 3050, Qatar; h Center for Integrated Bio & Nano Systems, University of Houston, Houston, TX 77204, USA; i Department of Biology and Biochemistry, University of Houston, Houston, TX 77204, USA; j Infectious Disease Epidemiology Group, Weill Cornell Medicine-Qatar, Cornell University, Doha, Qatar; k World Health Organization Collaborating Centre for Disease Epidemiology Analytics on HIV/AIDS, Sexually Transmitted Infections, and Viral Hepatitis, Weill Cornell Medicine-Qatar, Cornell University, Doha, Qatar; l Department of Population Health Sciences, Weill Cornell Medicine, Cornell University, New York, NY, USA

**Keywords:** Seroprevalence, Hepatitis E, HEV, Workers, Infectious disease

## Abstract

**Background:**

The rapid growth of Qatar in the last two decades has attracted a large influx of immigrant craft and manual workers (CMWs) seeking employment in jobs associated with food handling, domestic service, and construction. Nearly 60 % of Qatar's population are expatriates CMWs, including many from hyperendemic countries for HEV. Thus, estimating the seroprevalence of HEV in Qatar and understanding its epidemiology is essential for public health efforts to control HEV transmission in Qatar.

**Methods:**

Blood samples from 2670 CMWs were collected between 2020 and 2021. All samples were tested for HEV-IgG antibodies. Positive HEV-IgG samples were tested for HEV-IgM antibodies, and those positives were also tested for viral antigens using an HEV-Ag ELISA kit and HEV-RNA by RT-PCR to confirm current HEV infections.

**Results:**

The seroprevalence of HEV-IgG was 27.3 % (729/2670; 95 % CI: 25.6–29.0). Of those HEV-IgG positive, 8.23 % (60/729; 95 % CI: 6.30–10.5) were HEV-IgM positive. Of the IgM-positive samples, 2 were HEV-RNA positive (3.39 %; 95 % CI: 0.40–11.7), and 1 was HEV-Ag positive (1.69 %; 95 % CI: 0.04–9.09). In addition, HEV-IgG seroprevalence was associated with age and nationality, with the highest seroprevalence in participants from Egypt (IgG 60.0 %; IgM 5.56 %), Pakistan (IgG 59.0 %; IgM 2.24 %), Nepal (IgG 29.3 %; IgM 2.70 %), Bangladesh (IgG 27.8 %; IgM 2.45 %), and India (IgG 23.9 %; IgM 2.43 %).

**Conclusion:**

In this study, we showed that the seroprevalence of HEV among CMWs was slightly higher than what was previously reported among the urban population in Qatar (2013–2016).

## 1 Introduction

Hepatitis E virus (HEV) is a small, single-stranded RNA, non-enveloped, icosahedral virus. HEV has at least four distinct genotypes. Genotypes 1 and 2 have been found only in humans. Genotypes 3 and 4 circulate in animals and occasionally infect humans. HEV is transmitted via the fecal-oral route, mainly through contaminated water. However, other modes of transmission have recently been reported, such as vertical transmission in utero and blood donation. Acute infection from HEV can range from asymptomatic infection to acute hepatitis that is usually self-limiting and resolves within 2–6 weeks [1,2]. However, severe infections resulting in liver failure and death can occur in vulnerable groups such as pregnant women, immunocompromised individuals, and those with preexisting liver disease. Although the overall mortality rate is 1 %, it rises up to 20–25 % in pregnant women infected with HEV during the third trimester [1,[3], [4], [5]]. Typically, HEV is diagnosed through the identification of HEV antibodies using enzyme immunoassays (ELISA) and the identification of HEV-RNA in blood and fecal samples using real-time polymerase chain reaction (RT-PCR).

According to the National Institutes of Health (NIH), HEV is the most common, yet least diagnosed, cause of acute viral hepatitis worldwide [[6], [7], [8]]. Every year, an estimated 20 million infections occur globally, resulting in more than 3 million symptomatic cases and about 70,000 HEV-related fatalities [9,10]. This is likely underestimated due to a lack of testing and reporting [6,11,12]. HEV infection is reported worldwide, but it is more common in low- and middle-income countries owing to poor sanitation, and frequent outbreaks can occur from the consumption of fecally contaminated water [4,8,[13], [14], [15], [16]]. HEV remains a significant public health problem, with one-third of the global population residing in HEV-endemic regions [8]. In the last decades, HEV has been responsible for almost all water-borne outbreaks and most sporadic acute hepatitis incidence [17,18].

It is noteworthy that an effective HEV vaccine has been approved in multiple countries, demonstrating high efficacy against HEV while maintaining favorable tolerability [[18], [19], [20]]. This vaccine holds considerable promise in providing robust protection, especially for high-risk populations. The integration of vaccination initiatives, particularly among susceptible groups, plays a crucial role in preventing HEV infections and associated complications.

Qatar has a unique socio-demographic structure. Nearly, 60 % of the population are expatriate craft and manual workers CMWs, typically working in mega development projects [[21], [22], [23], [24], [25], [26], [27]]. Most CMWs come from highly HEV-endemic countries such as India, Nepal, Bangladesh, Sudan, and Egypt. For instance, our previous study reported that the seroprevalence of HEV among blood donors of some of these nationalities is more than 3-fold higher than that of the native Qatari population [28]. In recent years, more evidence for the direct person-to-person transmission of HEV has accumulated, and over 80 % of cases originated from families with multiple infected patients [29,30]. We hypothesized that inadequate sanitation, poor hygiene, and congregate living may enhance the risk of HEV infection among the CMWs. 1) determine the seroprevalence of HEV-IgG among CMWs in Qatar as an indicator of past or recent HEV exposure. 2) investigate the prevalence of recent HEV infection among CMWs by analyzing the presence of HEV-IgM, HEV-RNA, and HEV-Ag in IgG-positive individuals. We believe that our findings will be essential to inform strategies to limit the spread of HEV in the community and to improve the health of these populations.

## 2 Materials and methods

### 2.1 Study design and ethical compliance

2670 unidentified serum samples were collected from the Qatar Biobank for this study. These samples were collected as part of the national cross-sectional study to assess the seroprevalence of Severe acute respiratory syndrome coronavirus 2 (SARS-CoV-2) among CMWs between July 26 and September 09, 2020. The samples used in this study are the same we had used in our previous publications. For more information about the sampling process the readers are advised to refer to these studies [31,32]. To optimize the sample representativeness of the wider CMWs population without a comprehensive list, we devised a sampling strategy based on an analysis of the registered users’ database of the Qatar Red Crescent Society (QRCS), the main provider of primary health care for CMWs in the country. QRCS operates 4 geographically distributed centers that were specifically designed to cater to the CMW population across the country. These centers were established over a decade ago and are well known by CMWs, operate long working hours (3 run >24 h and 1 runs >16 h), are located in regions where workers live, and provide services that are free of charge or heavily subsidized for enhanced accessibility and affordability. The probability distribution of CMWs by age and nationality in the QRCS database was cross-checked and found to be similar to that of the Ministry of Interior database of expatriate residents [33]. All samples were anonymously collected without any identifying information other than sex, age, and nationality. Written informed consent was collected from all study participants. This study was approved by IRB at Qatar University (QU-IRB 1558-EA/21).

### 2.2 Laboratory testing

#### 2.2.1 Detection of HEV-IgG, HEV-IgM, and HEV-Ag

The HEV Wantai kits are one of the most widely used commercial ELISA kits in seroprevalence studies. All serum samples were screened for HEV-IgG using the Wantai HEV-IgG kit (Catalog # WE-7196, Wantai Co., Ltd., Beijing, China). In addition, all HEV-IgG positive samples were screened for HEV-IgM using the Wantai HEV-IgM kit (Catalog # WE-7296, Wantai Co., Ltd., Beijing, China). All positive HEV-IgM samples were tested for HEV-Ag using the Wantai HEV-Ag ELISA kit (Catalog # WE-7596, Wantai Co., Ltd., Beijing, China), except for one sample, which was not tested due to insufficient volume. All tests were conducted according to the manufacturer's instructions.

### 2.3 Detection of HEV-RNA using real-time PCR

All HEV-IgM positive samples, except for one sample due to insufficient volume, were tested for HEV-RNA using RT-PCR. For RNA extraction, QIAamp® Viral RNA Mini Kit (Qiagen; Hilden, Germany) was used according to the manufacturer's instructions. Using the QuantStudioTM 6 Flex Real-Time PCR instrument, reverse transcription and amplification of HEV RNA were carried out using 10 μL of the extracted RNA following the instructions provided by the manufacturer of the AmpliCube HEV RT-PCR kit (Mikrogen, Neuried, Germany). Samples with CT values more than 40 were considered negative as per the kit specification.

### 2.4 Statistical analysis

Seroprevalence and 95 % CI were estimated based on HEV-IgG antibody-positive results. 2562 participants had their ages and nationalities recorded, while the remaining 108 were marked as missing due to a lack of information. Descriptive statistics were used to explore the characteristics of the study samples. For the bivariate analysis, we employed crosstabs and Pearson's chi-square to investigate the relationship between the HEV status and the putative individual-related variables. In addition, a multivariate logistic regression model was employed to investigate the association between the presence or absence of HEV antibodies and independent factors, including age and nationality. All statistical analysis was performed using GraphPad Prism software (Version 8.2.1. San Diego, CA, USA).

## 3 Results

### 3.1 Characteristics of the study cohort

**Fig. 1:**
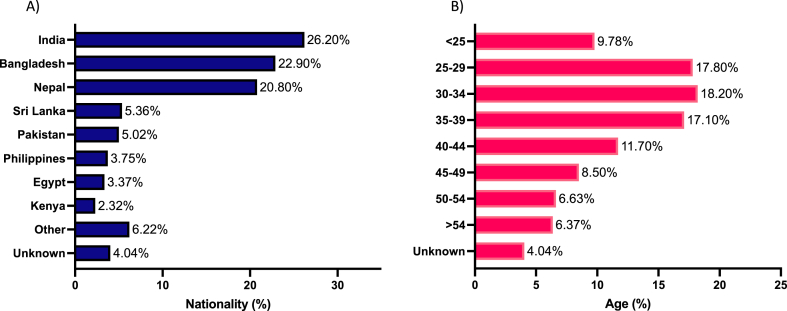
A) representative figure of the study cohort by nationality. B) representative figure of the study cohort by age groups.

The mean age of participants was 36.7 ± 10.5 ranging from 18 to 80 years old. 50 % of the participants were aged between 25 and 39 years (Table 1). The relative distribution by nationality showed that the largest percentage of participants were from India (26.2 %), followed by Bangladesh (22.9 %) and Nepal (20.8 %), as shown in Fig. 1 A and B.

**Table 1:** Demographic characteristics of participants in this study (n = 2670).

Characteristic	Number (%)
Nationality	
India	699 (26.2 %)
Bangladesh	612 (22.9 %)
Nepal	556 (20.8 %)
Sri Lanka	143 (5.36 %)
Pakistan	134 (5.02 %)
Philippines	100 (3.75 %)
Egypt	90 (3.37 %)
Kenya	62 (2.32 %)
Other	166 (6.22 %)
Unknown	108 (4.04 %)
Age	
<25	261 (9.78 %)
25–29	475 (17.8 %)
30–34	485 (18.2 %)
35–39	456 (17.1 %)
40–44	311 (11.7 %)
45–49	227 (8.50 %)
50–54	177 (6.63 %)
>54	170 (6.37 %)
Unknown	108 (4.04 %)

### 3.2 HEV-IgG, HEV-IgM, HEV-Ag, and RT-PCR

The HEV-IgG seroprevalence was 27.3 % (n = 729; 95 % CI (25.6–29.0)) among CMWs. Of those HEV-IgG positive, 8.23 % (60/729; 95 % CI: 6.30–10.5) were HEV-IgM positive. The estimated seroprevalence of HEV-IgM among the 2670 samples collected from the CMWs is 2.25 % (n = 60). Table 2 shows the frequencies of serological markers of HEV among CMWs in Qatar.

**Table 2:** Serological markers of HEV among CMWs in Qatar.

Serologic Markers	Total No.	Pos No.	Neg No.	Pos % (95 CI)	Neg % (95 CI)
Anti HEV IgG	2670	729	1941	27.3 (25.6–29.0)	72.7 (70.9–74.4)
Anti HEV IgM	729^a^	60	669	8.23 (6.30–10.5)	91.8 (89.5–93.7)
HEV RNA (PCR)	59^b^	2	57	3.39 (0.40–11.7)	96.6 (88.3–99.6)
Anti HEV Ag	59^b^	1	58	1.69 (0.04–9.09)	98.3 (90.9–100)

a Only HEV-IgG positive samples were tested for HEV-IgM.

b Only HEV-IgM positive samples were tested HEV-RNA and HEV-Ag. 1 sample was not tested due to insufficient volume.

Further, all positive HEV-IgM samples (except one because of insufficient volume; n = 59) were tested for HEV-RNA and HEV-Ag. Only two samples (2/59; 3.39 %) were HEV-RNA positive. These positive RT-PCR samples belonged to patients from Nepal and India. In addition, only one sample tested positive for HEV-Ag, which belonged to a patient from Egypt.

### 3.3 HEV-RNA and serological markers association with age and race

A statistically significant difference was found in the seropositive rate of HEV-IgG across different age groups, while no significant difference was observed in the seropositive rate of HEV-IgM among them. Similarly, a statistically significant difference was found in the seropositive rate of HEV-IgG across different nationalities, while no significant difference was observed in the seropositive rate of HEV-IgM among them.

**Fig. 2:**
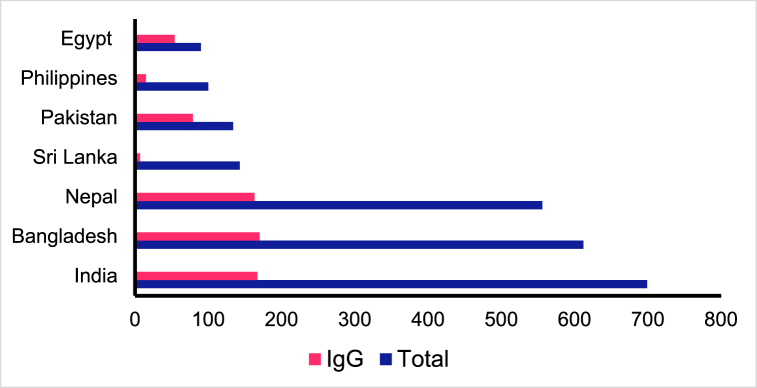
Seroprevalence of HEV-IgG among CMWs in Qatar. Total indicates the total number of participants.

HEV-IgG seropositivity was significantly associated (*p* < 0.0001) with age among the CMWs population in Qatar (Table 3). HEV-IgG seropositivity increased significantly with age and peaked at about 40.0 % among those aged >54 year-olds. In addition, HEV-IgG seropositivity was significantly associated (*p* < 0.0001) with nationality among the CMWs population (Table 3). HEV-IgG seroprevalence was 59.0 %, 60.0 %, 29.3 %, 27.8 %, and 23.9 % in participants from Pakistan, Egypt, Nepal, Bangladesh, and India, respectively (Table 3 and Fig. 2).

**Table 3:**
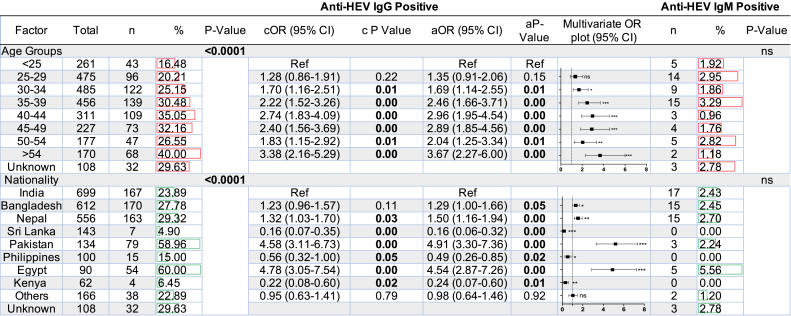
Analysis of potential associated factors for HEV status among CMWs.

The Bivariate analysis showed significant difference in the seropositive rate of HEV-IgG between different age groups and nationalities. Therefore, the multivariate odds ratio was calculated. On the other hand, no significant difference was observed between the seropositivity of HEV-IgM and different age groups or nationalities. Bold indicates significance at P < 0.05, cOR: Crude odds ratio, aOR: Adjusted odds ratio. NA: Not applicable.

## 4 Discussion

HEV remains a serious public health concern in the Middle East, where its prevalence ranged from 2.0 % to 37.5 % and was greater among males than females [15]. In this study, we assessed the seroprevalence of HEV among the CMWs population in Qatar. The seroprevalence of HEV-IgG was 27.3 % (729/2670; 95 % CI: 25.6–29.0). Of those HEV-IgG positive, 8.23 % (60/729; 95 % CI: 6.30–10.5) were HEV-IgM positive. The high seroprevalence in Qatar was expected owing to the country's diverse population, the vast majority of whom originates from highly HEV endemic countries such as Egypt and the Indian subcontinent, including India, Nepal, and Bangladesh [34]. Therefore, it is likely that most of the HEV cases in Qatar were imported either via a large inflow of migrant workers or through frequent travel to these countries.

We also believe that the close contact due to living in a labour campus has a great impact on the seroprevalence of HEV among the CMWs in Qatar. The HEV seroprevalence among CMWs was determined to be 27.3 %, exceeding the previously reported rate among urban residents in Qatar during 2013–2016 [28]. In this context, the seroprevalence of HEV-IgG among Egyptian (60.0 %) and Indian (23.9 %) CMWs was almost 1.6 fold higher than the urban population from both nationalities, 38.8 % and 15.1 %, respectively [28]. Similarly, the CMWs from Philippines (15.0 %) and Pakistan (59.0 %) showed higher HEV seroprevalence compared to the urban residents from the same nationalities, 12.1 % and 40.9 %, respectively [28].

HEV seroprevalence in Qatar is comparable to that of its Gulf countries neighbors. Studies from Saudi Arabia showed a prevalence of HEV ranging from 7.4 % to 18.7 % [[35], [36], [37], [38]]. In addition, a study conducted in Dubai, United Arab Emirates, between 2006 and 2007 indicated that 40 % of acute hepatitis patients had HEV infection [39]. Moreover, a study in Oman reported that 16.4 % of confirmed viral hepatitis cases were positive for HEV [40]. Furthermore, Al-Nasrawi et al., reported that 38.1 % of patients presenting with jaundice were found to be HEV-IgM positive in Iraq [41]. Sudanese had the greatest seroprevalence of HEV [13,42,43]. Since the fecal-oral route is the primary mode of HEV transmission, Sudanese and South Sudanese people are exposed to polluted water, resulting in many HEV outbreaks [13,42,43]. Outbreaks of HEV have been reported in Sudan since 2015 due to appalling living conditions, including inadequate water, sanitation, and hygiene [44]. Similar arguments are applied to Pakistan and Egypt. A study conducted in Egypt showed that the HEV seroprevalence exceeded 60 % in all age groups [45,46]. The WHO published a systematic review reporting many outbreaks of HEV infection in many countries [45]. As large as these figures are, this likely represents a gross underestimate of the actual global disease burden [47].

Similar to earlier published data [[28], [29], [30], [31]], the HEV-IgG seropositivity increased significantly with age in our study. This may be explained by the long-term persistence of the IgG antibody in human blood after HEV infection [13,17], the longer length of exposure for those in older age groups, and probably recurrent exposure over time in hyperendemic nations.

In this study, HEV-IgM was detected in 60 CMWs, of which viremia was confirmed in only 2 samples by RT-PCR, suggesting a viremia ratio of 1:1335 among CMWs in Qatar (3.51 %). However, this ratio could be underestimated as HEV-IgM stays detectable during the convalescence period (3–6 months after infection), where RNA levels usually decline [1,5,48]. Another reason could be that HEV-IgM might not have reached detectable levels at the early stages of infection (window period), where RNA usually peaks [48]. Therefore, many viremic cases may have been missed by conducting RT-PCR on HEV-IgM-positive samples only. Most importantly, samples may not have been transported and stored adequately to protect viral RNA; whereas antibodies are very stable, the viral RNA is much less stable. In this context, 54 samples in our study showed positive HEV-IgM and HEV-IgG but negative RT-PCR results. These 54 patients could be in an early convalescence stage, where RNA disappeared, or could be due to the low level of HEV-RNA in their serum [49]. Therefore, relying on antibody detection alone could lead to misdiagnosis of acute HEV in many infected cases. Several studies have indicated that RNA detection of HEV could be of particular importance, especially in cases that involve blood donation or transfusion. Accordingly, HEV RNA detection is the gold standard for uncovering viremia [48,50]. It is worth noting that HEV-RNA may be detected for a longer duration in stool samples compared to blood during the acute stage [51,52]. Therefore, testing fecal samples from these patients might be essential for an accurate diagnosis.

Zhang et al. have indicated that HEV-Ag in macaques became detectable in the serum at almost the same time as HEV-RNA in faeces [53]. Others suggested that HEV-Ag detection should be a valuable tool for diagnosing acute HEV, particularly in the window period before seroconversion to anti-HEV [53,54]. To our knowledge, Wantai Ag-ELISA is the only commercial assay currently present in the market for diagnosing HEV Ag. In our previous study, we assessed the performance of Wantai Ag-ELISA for diagnosing acute HEV [55]. Although HEV-Ag was very specific (100 %), its sensitivity was poor (36.7 %), suggesting that the Wantai HEV-Ag might not be very useful to be used as a single screening assay [55]. In this study, only one HEV-IgM positive sample tested positive using Wantai Ag-ELISA; however, this sample tested negative by RT-PCR. Due to the high specificity of the assay (100 %), positive HEV-IgG and HEV-IgM, we could suggest that this sample was false negative by the RT-PCR. False negative RT-PCR test results could occur due to RNA degradation, poor RNA extraction, early convalescence stage, where RNA disappears or technical errors [49,56].

It is worth acknowledging that the sensitivity of the Wantai HEV-Ag assay may vary depending on the disease progression. While it generally exhibits higher sensitivity in diagnosing acute HEV, its efficacy might be somewhat reduced when diagnosing self-limiting infections [57]. The detectability of HEV Ag is influenced by variables such as the plasma viral load and levels of liver enzymes. Additionally, a negative HEV-Ag outcome may occur if the sample was collected during the convalescence stage of the infection.

Previous research has demonstrated the practical utility of the Wantai HEV-Ag assay as a reliable diagnostic tool in clinical settings where molecular assays may not be readily accessible [[58], [59], [60]]. Additionally, the Wantai HEV-Ag assay exhibits the capability to differentiate between acute and chronic HEV-3 infections [61], with the serum levels of HEV Ag potentially serving as a predictor of the likelihood of HEV chronicity, especially among immunocompromised patients. Furthermore, acute HEV infection can lead either to a self-limiting disease course or progression to fulminant hepatic failure, with delayed initiation of anti-HEV therapy (e.g., ribavirin) contributing to morbidity [62]. These finding collectively underscore the multifaceted applications of the Wantai HEV-Ag assay, expanding its role beyond diagnostics to aiding clinical decision-making and interventions for HEV infections.

There are several limitations in our study. Information about potential risk factors such as education, socioeconomic status, period of residency in Qatar, and travel history is not available. The findings could not be generalized to the total population, as the cohort in our study consisted of only male participants. Finally, we could not test all collected samples using RT-PCR, and we did only for the HEV-IgM positive samples, which could underestimate the true prevalence of HEV among the CMWs in Qatar.

## Funding

This report was made possible by GSRA8-L-1-0501-21022 and NPRP13S-0128-200185 from the Qatar National Research Fund (a member of 10.13039/100007458Qatar Foundation). Laith Abu-Raddad and Houssein H. Ayoub would like to acknowledge receiving funds from NPRP12S-0216-190094 from the Qatar National Research Fund. The funders had no role in study design, data collection, analysis, the decision to publish, or the preparation of the manuscript. The statements made herein are solely the responsibility of the authors.

## Declaration

This study was approved by IRB at 10.13039/501100004252Qatar University (QU-IRB 1558-EA/21).

## Data availability statement

Data included in article/supp. material/referenced in article.

## CRediT authorship contribution statement

**Nadin Younes:** Writing – review & editing, Writing – original draft, Visualization, Validation, Software, Conceptualization. **Hadi M. Yassine:** Writing – review & editing, Visualization, Validation, Supervision, Project administration, Methodology, Conceptualization. **Parveen Banu Nizamuddin:** Methodology. **Katerina Kourentzi:** Writing – review & editing, Visualization. **Patrick Tang:** Writing – review & editing, Validation. **Houssein H. Ayoub:** Writing – review & editing, Visualization. **Makiyeh Khalili:** Writing – review & editing, Validation, Investigation, Formal analysis, Data curation. **Peter V. Coyle:** Writing – review & editing, Validation, Project administration, Investigation, Formal analysis, Data curation. **Dmitri Litvinov:** Writing – review & editing, Visualization. **Richard C. Willson:** Writing – review & editing, Visualization. **Laith J. Abu-Raddad:** Writing – review & editing, Visualization, Validation. **Gheyath K. Nasrallah:** Writing – review & editing, Writing – original draft, Visualization, Supervision, Resources, Project administration, Investigation, Funding acquisition, Data curation, Conceptualization.

## Declaration of competing interest

The authors declare the following financial interests/personal relationships which may be considered as potential competing interests:

Dmitri Litvinov has patent licensed to FemtoMag, Inc. Corresponding author is an associate editor at Heliyon-Infectious Disease Journal. GKN.
